# Development of a bacterial cellulose-based hydrogel cell carrier containing keratinocytes and fibroblasts for full-thickness wound healing

**DOI:** 10.1038/s41598-018-21174-7

**Published:** 2018-02-13

**Authors:** Evelyn Yun Xi Loh, Najwa Mohamad, Mh Busra Fauzi, Min Hwei Ng, Shiow Fern Ng, Mohd Cairul Iqbal Mohd Amin

**Affiliations:** 1 Faculty of Pharmacy, Universiti Kebangsaan Malaysia, Jalan Raja Muda Abdul Aziz, Kuala Lumpur, 50300 Malaysia; 2 Tissue Engineering Centre, Universiti Kebangsaan Malaysia Medical Centre, Jalan Yaacob Latif, Bandar Tun Razak, Kuala Lumpur, 56000 Malaysia; 3 Present Address: Faculty of Pharmacy, Cyberjaya University College of Medical Sciences, 3410, Jalan Teknokrat 3, Cyber 4, Cyberjaya, Selangor, 63000 Malaysia

## Abstract

Bacterial cellulose (BC)/acrylic acid (AA) hydrogel has successfully been investigated as a wound dressing for partial-thickness burn wound. It is also a promising biomaterial cell carrier because it bears some resemblance to the natural soft tissue. This study assessed its ability to deliver human epidermal keratinocytes (EK) and dermal fibroblasts (DF) for the treatment of full-thickness skin lesions. *In vitro* studies demonstrated that BC/AA hydrogel had excellent cell attachment, maintained cell viability with limited migration, and allowed cell transfer. *In vivo* wound closure, histological, immunohistochemistry, and transmission electron microscopy evaluation revealed that hydrogel alone (HA) and hydrogel with cells (HC) accelerated wound healing compared to the untreated controls. Gross appearance and Masson’s trichrome staining indicated that HC was better than HA. This study suggests the potential application of BC/AA hydrogel with dual functions, as a cell carrier and wound dressing, to promote full-thickness wound healing.

## Introduction

The skin is involved in many vital functions such as control of body temperature, keeping a balance of water and electrolyte, and detecting sensation. It is the main protective barrier of the body. Thus, when these functions are lost through skin injury such as burns, ulcers, or trauma, instantaneous coverage is required to assist repair and restore its function^1,2^. Wound dressings have long been used to aid healing by providing a physical barrier against infection, maintaining a moist wound environment, and absorbing exudates around the wound, but it cannot restore lost tissue in full-thickness wounds^3^. Currently, the ‘gold standard’ for treatment of full-thickness injuries is split-skin grafting (SSG), but there are various disadvantages, including inadequate availability of healthy skin, scar formation, and risk of infection^4^. Besides, cell-based skin regenerative products in the market present some limitations such as extremely high cost, the use of xenogeneic or allogeneic cells that carries the risk of rejection, and longer cell culture period^1,4,5^. These drawbacks led to the development of new strategies to deliver non-cultured autologous skin cells to the wound site using biomaterials such as hydrogel as a cell carrier for immediate treatment.

Hydrogels are three-dimensional (3D) hydrophilic, crosslinked polymeric networks capable of taking in a large amount of water causing it to swell while maintaining their 3D structure without dissolving^6^. Due to their high-water content, hydrogels are appealing as a scaffold because they resemble natural soft tissue, which includes the skin^7^. Many different materials in different forms have been investigated as scaffolds for skin tissue regeneration. Nicholas and co-workers described a combination of pullulan, a polysaccharide with antioxidant properties with gelatin, a collagen derivative able to absorb high amounts of water, to form a hydrogel acting as a bilayer skin substitute^5^. In another study^8^, a freeze-dried scaffold consisting of genipin crosslinked sericin seeded with keratinocytes and fibroblasts was developed as a skin equivalent. Sericin is a component found in the cocoon of silkworm. It is antibacterial, antioxidant and non-toxic^8^. Besides, alginate hydrogel was used to encapsulate fibroblasts, while keratinocytes were cultured on the surface^9^. Alginate is similar in structure to natural glycosaminoglycan found in the extracellular matrix (ECM)^10^. Mazlyzam and colleagues employed autologous fibrin derived from plasma as a fully autologous skin substitute, eliminating the risk of rejection^11^.

Bacterial cellulose-based hydrogels are attractive materials for wound dressing application due to its hydrophilic properties, purity, ability to maintain appropriate moisture balance and flexibility in conforming to any contour of the wound forming a tight barrier between the wound and the environment, thus, preventing bacterial infections^12,13^. It also found its place in tissue engineering application because of its biocompatibility, non-toxic effects, porous structure, and good mechanical strength^12^. We previously characterized and evaluated the potential of bacterial cellulose/acrylic acid (BC/AA) hydrogel as a wound dressing for partial-thickness burn wound. The hydrogel retained adequate moisture content and displayed sufficient mechanical strength with high elasticity and flexibility, properties all in favor of a wound dressing^14,15^. Furthermore, *in vivo* studies demonstrated that the wound healing rate was faster in the BC/AA hydrogel treatment group than that in the no treatment group (negative control) and Intrasite Conformable^®^ hydrogel treatment group (positive control) based on gross appearance and histological evaluation^14^. Additionally, the BC/AA hydrogel did not induce skin irritation on rabbits or skin sensitivity on guinea pigs^15^.

Bacterial cellulose is non-biodegradable in the human body because of the absence of cellulase enzyme^16^. Based on this, the previous abovementioned potential scaffold properties, and promising results of our previous studies^14,15^, BC/AA hydrogel may act both as a cell carrier and a wound dressing. Besides, the most severe forms of injury require exogenous cells for skin tissue regeneration. Hence, in this study, we investigated the wound healing ability of the BC/AA hydrogel carrying human epidermal keratinocytes (EK) and dermal fibroblasts (DF) in a full-thickness wound model. The *in vitro* evaluation of cell attachment, viability, proliferation, cell migration, and cell transfer of BC/AA hydrogel were performed prior to evaluating skin wound healing efficiency in an animal model *in vivo*.

## Results and Discussion

### Cell attachment

**Figure 1:**
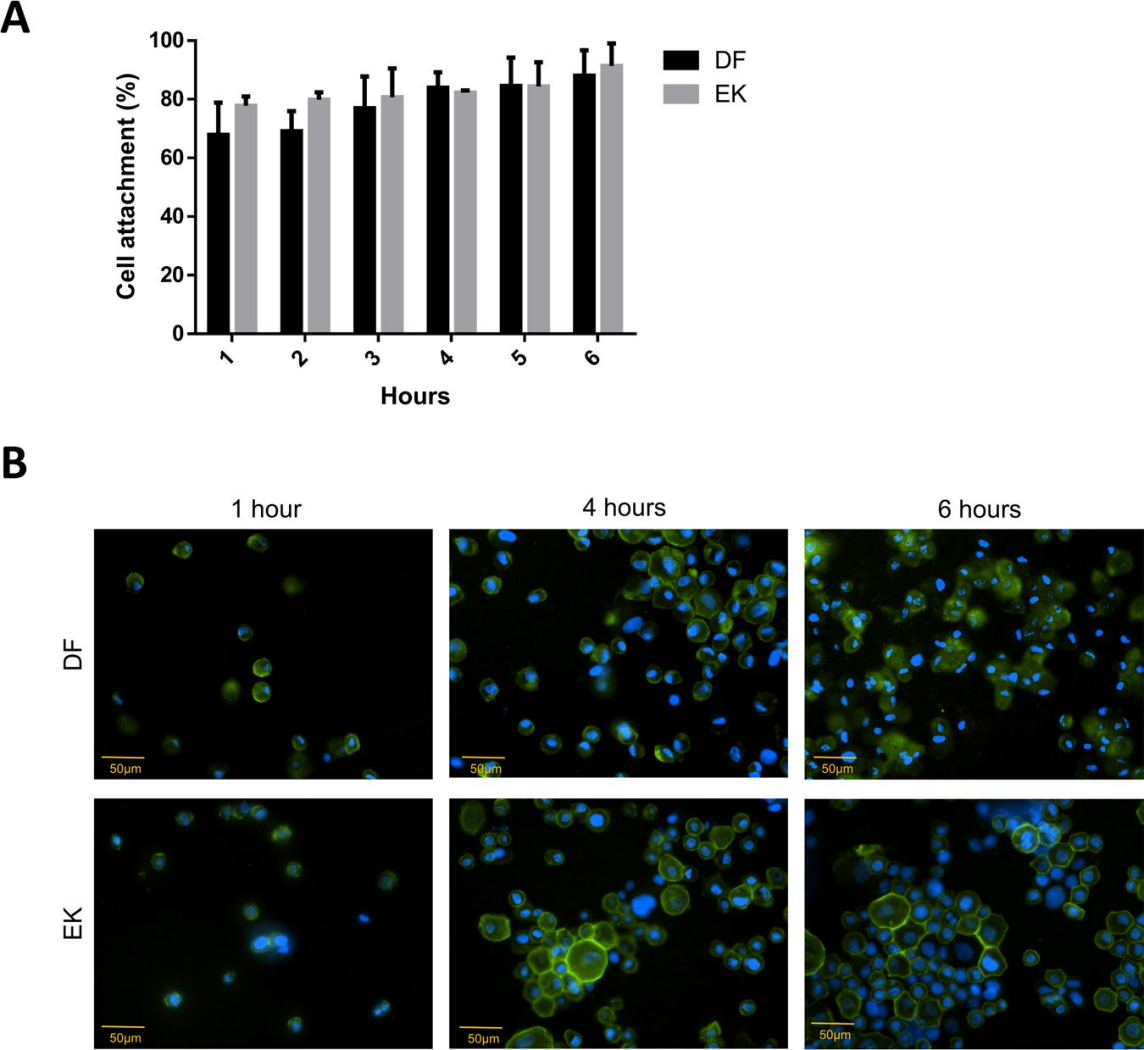
Cell attachment studies. (**A**) Percentage of dermal fibroblasts (DF) and epidermal keratinocytes (EK) attached on the hydrogel at 1 to 6 hours. No significant difference was observed between the 2 cell types (p > 0.05) (**B**) Phalloidin and DAPI staining for DF and EK at 1, 4 and 6 hours under 200× magnification.

The number of dermal fibroblasts (DF) and epidermal keratinocytes (EK) attached to the BC/AA hydrogel increased from 1 hour to 6 hours. This was also confirmed by the fluorescence image at 1 hour, 4 hours, and 6 hours (Fig. 1). No significant difference was observed between the two cell types in the number of cells attached at each time point. In fact, 84.0 ± 5.3% of DF and 82.3 ± 0.7% of EK attached on the hydrogel at 4 hours. These findings suggested that the hydrogel containing cells could be transferred to the wound site after a minimum of 4 hours as more than 80% of the cells have already attached.

Cell attachment findings on another type of scaffold by Huang *et al*.^18^ also demonstrated that the cell number for human keratinocytes and human foreskin fibroblasts seeded on collagen type I (COL-I) as a dermal scaffold and gelatin hydrogel as an epidermal scaffold gradually increased from 2, 6 and 24 hours. Cell attachment on any fabricated scaffold or cell carrier is a very essential initial step for subsequent events to occur, including survival, proliferation, and migration^19^. The BC/AA hydrogel showed excellent and rapid cell attachment within 4 hours where more than 80% of EK and DF successfully adhered onto the hydrogel. It is well known that hydrogel, which is hydrophilic in nature, has the potential to support cell attachment. A previous study indicated that hydrophilic surfaces enhanced fibroblasts attachment compared to hydrophobic surfaces^20^. Another possible reason for the excellent cell attachment is the larger surface area available for cells to adhere^8^ since the pore size, evaluated by Mohamed *et al*.^15^ was relatively small. Surface roughness is also known to facilitate cell attachment^21^. Faster cell adhesion is preferable to expedite the cell transfer on the wound site upon skin injury and to reduce the patient’s waiting time after being hospitalized.

### Cell viability, proliferation and morphology

**Figure 2:**
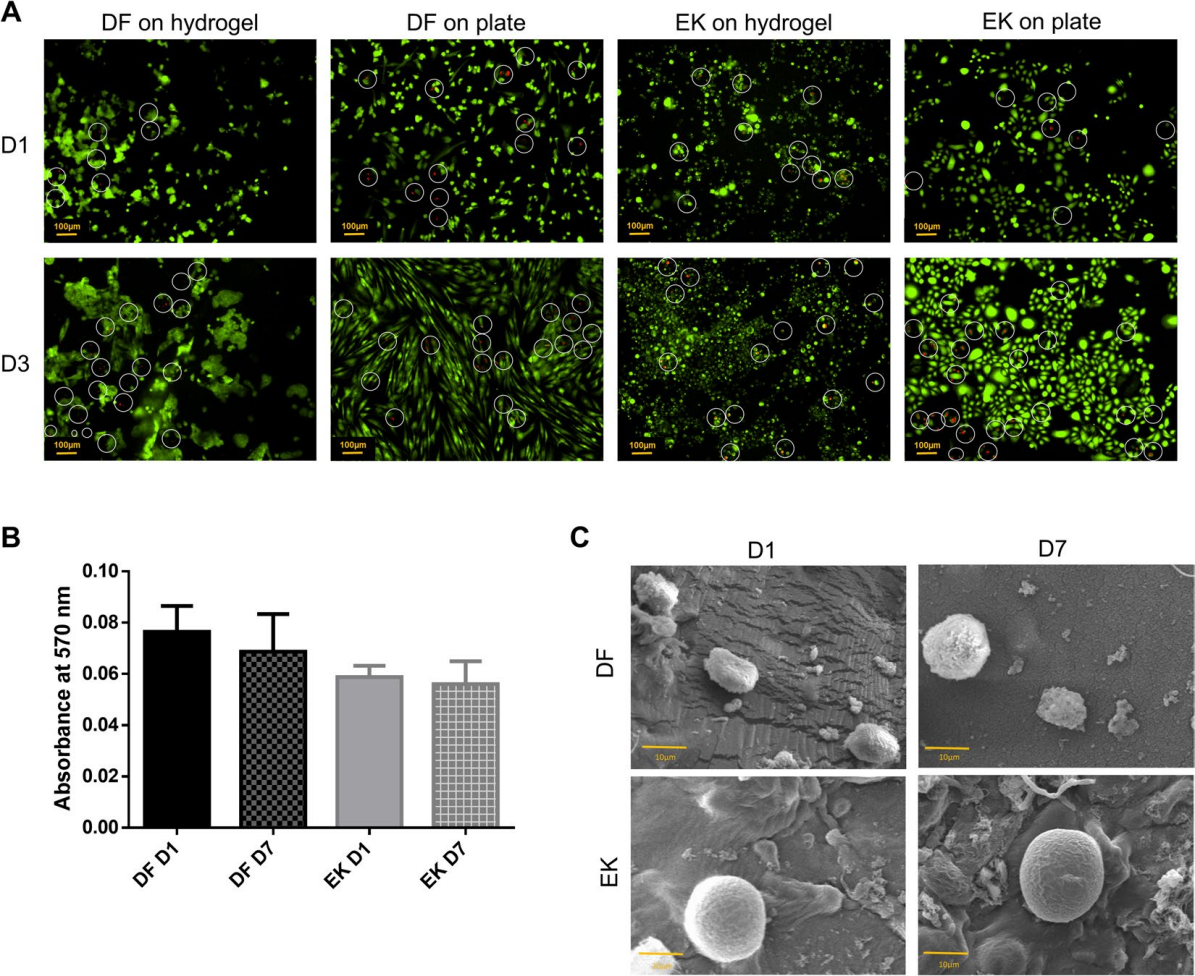
(**A**) Live (green) and dead (red) assay of dermal fibroblasts (DF) and epidermal keratinocytes (EK) on hydrogel and polystyrene cell culture plates at day 1 (D1) and day 3 (D3) under 100× magnification. The white line circle indicates the dead cells (**B**) Cell proliferation of DF and EK on the hydrogel at day 1 and day 7 (D7) quantified by using MTT assay. No significant difference was observed at D1 and D7 (p > 0.05) (**C**) SEM images of DF and EK on the hydrogel at day 1 and day 7 under 700× magnification and 13 kV.

The numbers of red EK and DF observed on hydrogel and on polystyrene culture plates at day 1 and 3 (Fig. 2A) were few and comparable, being mainly predominated by green EK and DF indicating that the hydrogel supports the viability of cells. Both types of cells seeded on the hydrogel showed similar trends when evaluated by using the MTT assay (Fig. 2B). Even though there was a slight reduction in the absorbance reading at day 7 compared to day 1, the reduction was not significant, demonstrating that the hydrogel maintains cell viability throughout the seven days.

A similar trend was also observed by Mohd Hilmi *et al*.^22^ on a different type of scaffold namely chitosan sponge, where the absorbance of DF remained constant throughout 14 days, even though DF were spindle shape based on micrographs. The 3D structure of BC/AA hydrogel seems inadequate to provide a suitable environment for cell proliferation probably due to its relatively smaller pore size, less porous and the possible presence of low acidic content inside the hydrogel despite various attempts to completely neutralize it^23,24^.

EK and DF cultured on the hydrogel under scanning electron microscope (SEM) demonstrated a round shape morphology from day 1 to day 7 with matrix produced at its surrounding (Fig. 2C), suggesting that the cells were not proliferating, consistent with the MTT findings (Fig. 2B). When EK and DF are grown on polystyrene culture plate, EK present a polygonal shape, while DF present a spindle shape^25^ as observed in Fig. 2A. The round shape of EK and DF shows that they do not extend and spread their lamellipodium or filopodium, structures that are commonly observed in migrating and dividing cells^26^. Although the BC/AA hydrogel only supports cell viability and not cell proliferation, such property is sufficient for its intended purpose to act as a cell carrier and is expected to transfer most of the cells from the hydrogel to the wound site within a few days.

### Cell migration and cell transfer

**Figure 3:**
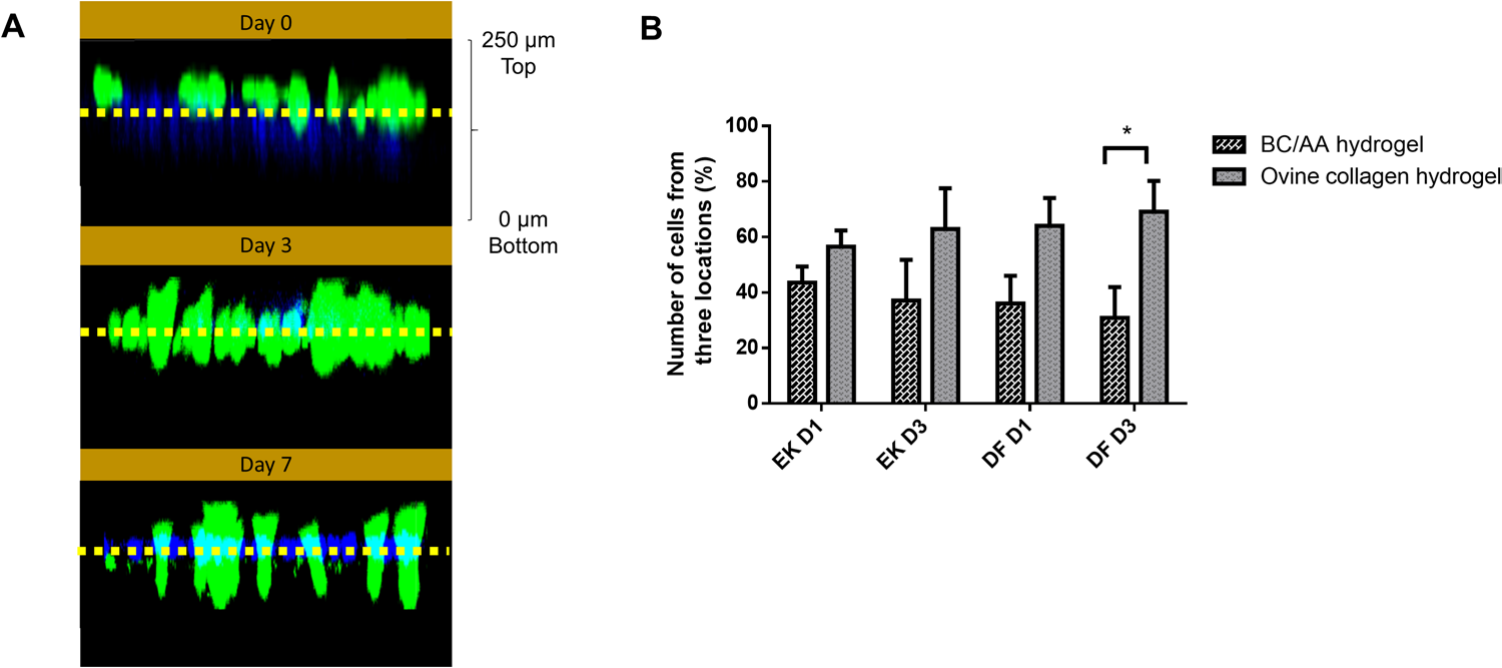
(**A**) 3D confocal imaging of cell migration (**B**) Number of epidermal keratinocytes (EK) and dermal fibroblasts (DF) on BC/AA hydrogel and ovine collagen hydrogel on day 1 (D1) and day 3 (D3) in the cell transfer study. The asterisk (*) represents a significant difference (p < 0.05) for DF D3 between BC/AA hydrogel and ovine collagen hydrogel.

In the cell migration study, EK and DF were stained in green and blue, respectively. No migration was detected as they remained at the top of the hydrogel from day 0 to day 7 (Fig. 3A). The morphological features of non-migrating cells were also supported by SEM as in Fig. 2C.

A previous study carried out by Sun *et al*.^27^ described that skin cells possess the capability to self-organize in a 3D scaffold, resulting in keratinocytes remaining on the top level and fibroblasts migrating downwards in the matrix. Thus, migration of DF to the bottom of the BC/AA hydrogel may be restricted by small pore size^8^. Mohamad *et al*.^15^ previously demonstrated that the pore size range of 406035 (AA/BC/electron beam dose) formulation was between 9.5–30.08 µm. An acceptable range for a scaffold is between 100–500 µm^28^. However, the aforementioned properties of the BC/AA hydrogel suggest its future application as a cell carrier, where the maintenance of adhered cells on the hydrogel’s surface greatly enhances cell transfer to the wound bed.

In the cell transfer study (Fig. 3B), 56.5 ± 5.9% of EK and 64.0 ± 10.1% of DF were transferred from BC/AA hydrogel to ovine collagen hydrogel (OCH) at day 1. The total cumulative transferred reached 62.9 ± 14.7% of EK and 69.1 ± 11.1% of DF on the OCH at day 3. On day 1, more than half of the cells were successfully transferred for both type of cells, but the number transferred decreased over time. The number of cells on the OCH was more than that on the BC/AA hydrogel for DF and EK on day 1 and day 3 with a significant difference for DF at day 3. The increase in the number of cells on the OCH is due to a combination of cell transfer and proliferation.

The results showed that the cells seeded on the hydrogel can be transferred to the wound site so that it may accelerate the wound healing process. The cell attachment study (Fig. 1) indicated that transfer at 4 hours after the cells were seeded on the hydrogel was the optimal time to place the hydrogel onto the skin, represented by the OCH in the *in vitro* study. The ECM of normal adult skin is made up of predominantly 80% of collagen type I (COL-I) and approximately 10% of collagen type III^29^. Previously, Fauzi and co-workers demonstrated that the collagen extracted from ovine tendon mainly consists of COL-I as the primary component, comparable to commercially available rat tail COL-I^17^. Thus, in the *in vitro* 3D model, the use of OCH to mimic the native ECM is deemed reasonable^30^. In this study, the cell transfer from BC/AA hydrogel to OCH may also be attributed to the dryness of the hydrogel with time due to airlifted interface conditions. Prior to *in vivo* application, these results, besides its excellent cell attachment within 4 hours, ability to keep cells viable, and its limited migration, support the use of BC/AA hydrogel as a cell carrier.

### Visual observation of wound closure

**Figure 4:**
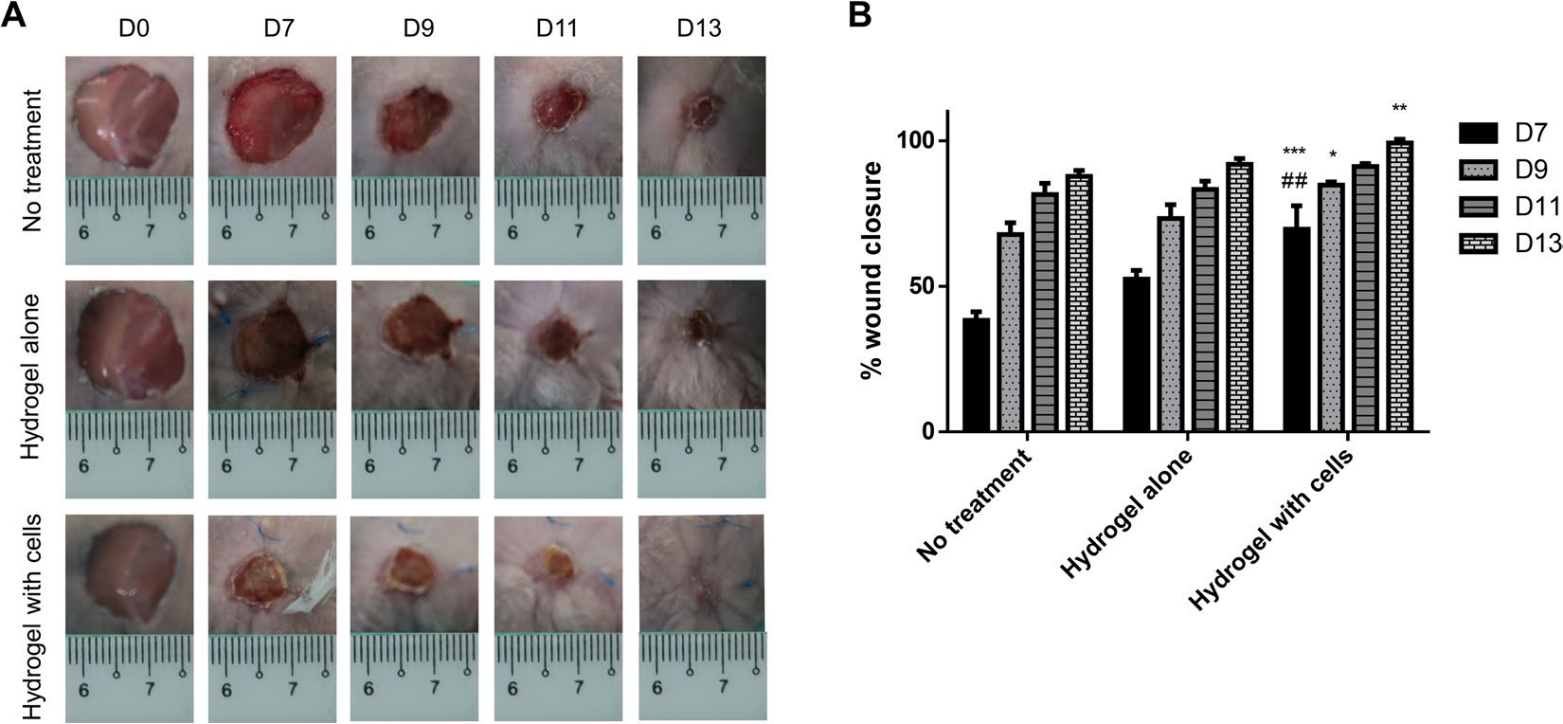
(**A**) Gross appearance of the wound and B) Percentage of wound closure from day 0 (D0) to day 13 (D13) for the different treatment groups. The asterisks (*), (**) and (***) represent significant differences (p < 0.05), (p < 0.01), and (p < 0.001), respectively compared to the no treatment group. The hashtag (##) represents a significant difference compared to the hydrogel alone group (p < 0.01) on the same day.

Visual observation in our *in vivo* animal model (Fig. 4A) showed that the wound area of all treatment groups was reduced over time, but the rate of healing differed. At day 13, the wound in mice treated with hydrogel with cells (HC) had completely healed compared to that in other groups with visible scab. Quantitative measurement of wound closure revealed that, at day 7, the percentage of wound closure was significantly higher in the HC treatment group [69.7 ± 8.0%] than that in both the hydrogel alone (HA) [52.5 ± 3.0%] and no treatment (NT) [38.4 ± 3.0%] groups (Fig. 4B). However, as the time progressed, only wound closure in the HC and NT groups at day 9 and 13 showed significant differences. Across all times, the wound closure between the HA and NT groups was comparable. At day 13, the wounds in mice treated with HC, HA, and NT were 99.2 ± 1.3%, 91.9 ± 2.0%, and 87.8 ± 2.0% healed, respectively. Besides, Wang and colleagues showed that the wound area of mice treated with bilayer skin substitute made of gelatin–chondrointin-6-sulfate–hyaluronic acid seeded with cells demonstrated almost complete healing in 2 weeks^31^.

During the *in vivo* experiment, the hydrogel detached from the mice skin after a week as it dried with time and no injury was detected at the wound area. This result is supported by the previous study performed by Mohamad and co-workers who demonstrated via the bioadhesiveness investigation that the 406035 (AA:BC:electron beam dose) hydrogel formulation was preferred as a wound dressing material due to easy removal and thus, does not cause pain and additional injury to the wound site^15^.

The combination of granulation tissue contraction and reepithelialization during the proliferative stage results in wound contraction. When sufficient matrix is formed, fibroblasts change to myofibroblasts where it contracts and pulls the wound edge together to decrease the size of the wound. Keratinocytes from the wound edges migrate and grow rapidly beneath the scab to restore the epidermis^32,33^. In this study, the wound closure results demonstrate that the wound heals significantly faster in the presence of HC, which is superior in promoting wound healing compared to no treatment. This clearly indicates that the addition of skin cells plays a significant role and can effectively improve the wound healing process. Besides, hydrogel, which is capable of maintaining the wound environment moist can also accelerate wound closure^32,34^. This may be associated with hydration and/or natural tissue fluids containing nutrients and growth factors being retained in the wound area^35^.

### Histological analysis

**Figure 5:**
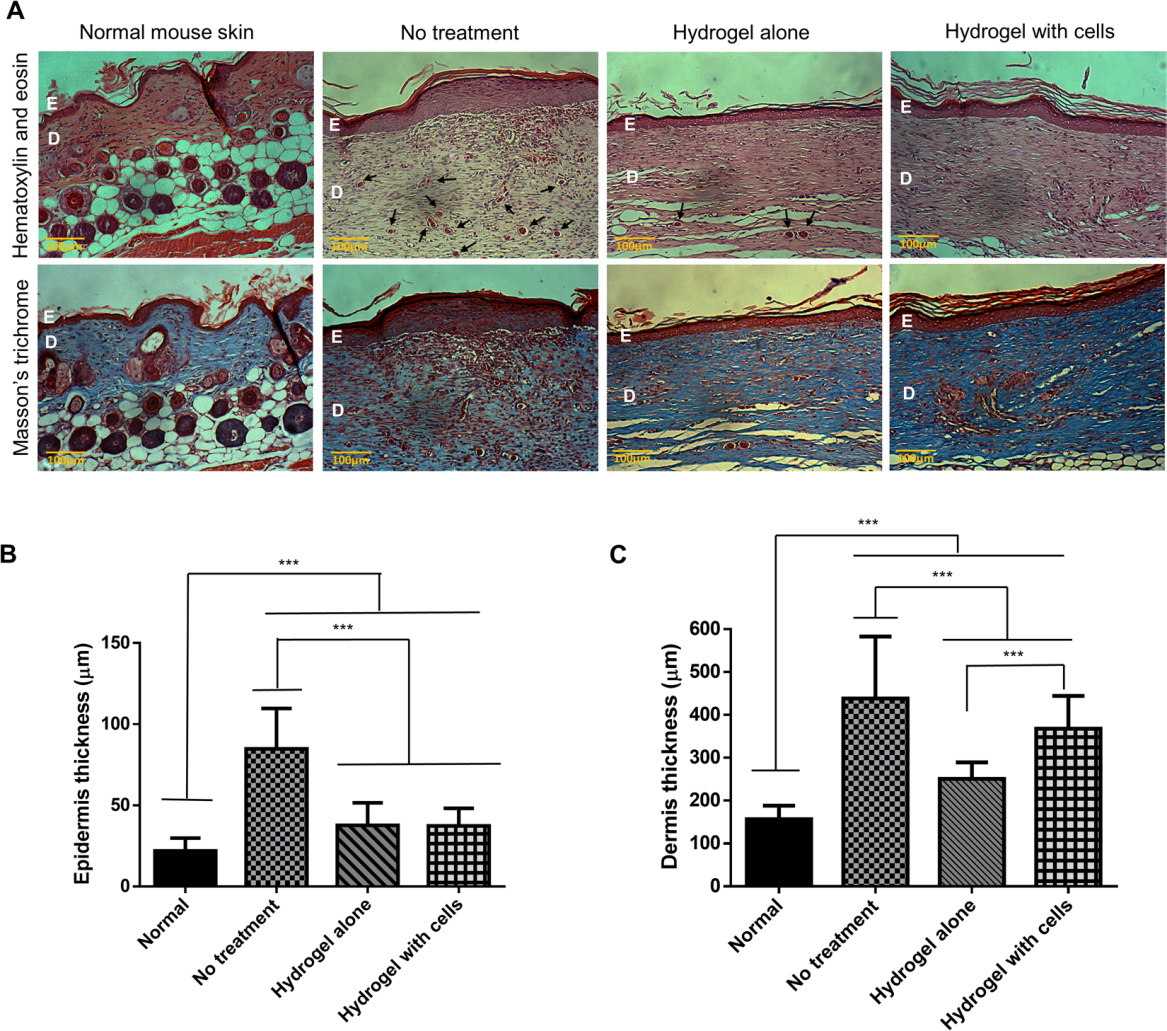
Histological analysis of the skin at day 13 post-treatment or no treatment. (**A**) H&E and Masson’s trichrome staining under 100× magnification. E refers to the epidermis layer; D refers to the dermis layer; arrow () refers to blood vessel (**B**) Thickness of the epidermis layer and (**C**) Thickness of the dermis layer of normal mouse skin compared to the different treatment groups. The asterisk (***) represents a significant difference (p < 0.001) between groups.

Hematoxylin and eosin (H & E) and Masson’s trichrome staining (Fig. 5A) showed that the wound of all groups reepithelialized by day 13 even though Fig. 4A showed the existence of crust or scab in the HA and NT groups. New epithelial cells formed as keratinocytes from the wound margin divided and migrated along the surface of the granulation tissue until they became a continuous layer below the scab^32^. This implies that reepithelialization occurred under the crust although the scab was still intact. Besides, the newly formed tissues of all the treated groups lack skin appendages such as glands and hair follicles compared to the normal skin. This is natural because skin appendages do not regenerate following skin injury^36^.

Many blood vessels were observed in the NT group. However, in the HA and HC treatment groups, the amount of blood vessels in the newly formed tissue subsided. This suggests that, at day 13, the wounds in the NT group were most likely still in the proliferative phase, while wounds in the HA and HC groups were seemingly already in the remodeling phase, during which the healing process is completing. During the proliferative stage, new blood vessels are formed from pre-existing vasculature, a process known as angiogenesis. This results in a temporary increase in the quantity of blood vessels at the injury site where they take part in the formation of granulation tissue and supply oxygen and nutrients to it. Over time, most of the vessels regress^29^. Furthermore, an increased number of cells was observed in the NT, HA, and HC groups as opposed to the normal skin since more cells were needed to regenerate the lost tissue upon wounding^37^.

Masson’s trichrome, which stains collagen fibers in blue, displayed more extensive collagen deposition in the HC group followed by the HA group, with the least amount of collagen fibers in the NT group. Collagen synthesized by fibroblasts is the fundamental component in supplying strength to the tissues and therefore, indicates that the HC treatment group presented a stronger skin compared to the other two groups^33^.

All wounded groups revealed significant thickening of the epidermal layer compared to the normal skin, with the skin of mice in the NT group being about four times thicker while the other two groups, which presented similar thickness was about twice that of the normal skin (Fig. 5B). Arai *et al*.^38^ investigated the cause of the temporary increased in epithelium thickness as a result of wounding and proposed that it is due to the rise in the number of keratinocytes brought about by increased proliferation and/or a delay in differentiation. Subsequent thinning is brought about by a sudden acceleration of differentiation into corneocytes to restore the epidermis to its normal thickness. Hence, this suggests that the HA and HC treatment groups were in a later phase of healing compared to the NT group.

As for the thickness of the dermal layer (Fig. 5C), the difference was significant across all groups. The dermis of the NT group was the thickest followed by HC, HA, and the normal skin. When collagen is deposited during the proliferative stage, it is disorganized and therefore, the dermis is thick. During the remodeling phase, collagen is rearranged into a more organized matrix achieved via cross-linking of collagen fibers^39^. This may explain why the dermis layer of all the injured groups is much thicker than that of the normal skin. It is also noteworthy that the cells supplied influenced the dermis thickness in comparison with HA. Regardless, the dermal layer of mice in the HA and HC treatment groups was more closely packed than that of mice in the NT group.

### Immunohistochemistry (IHC) staining

**Figure 6:**
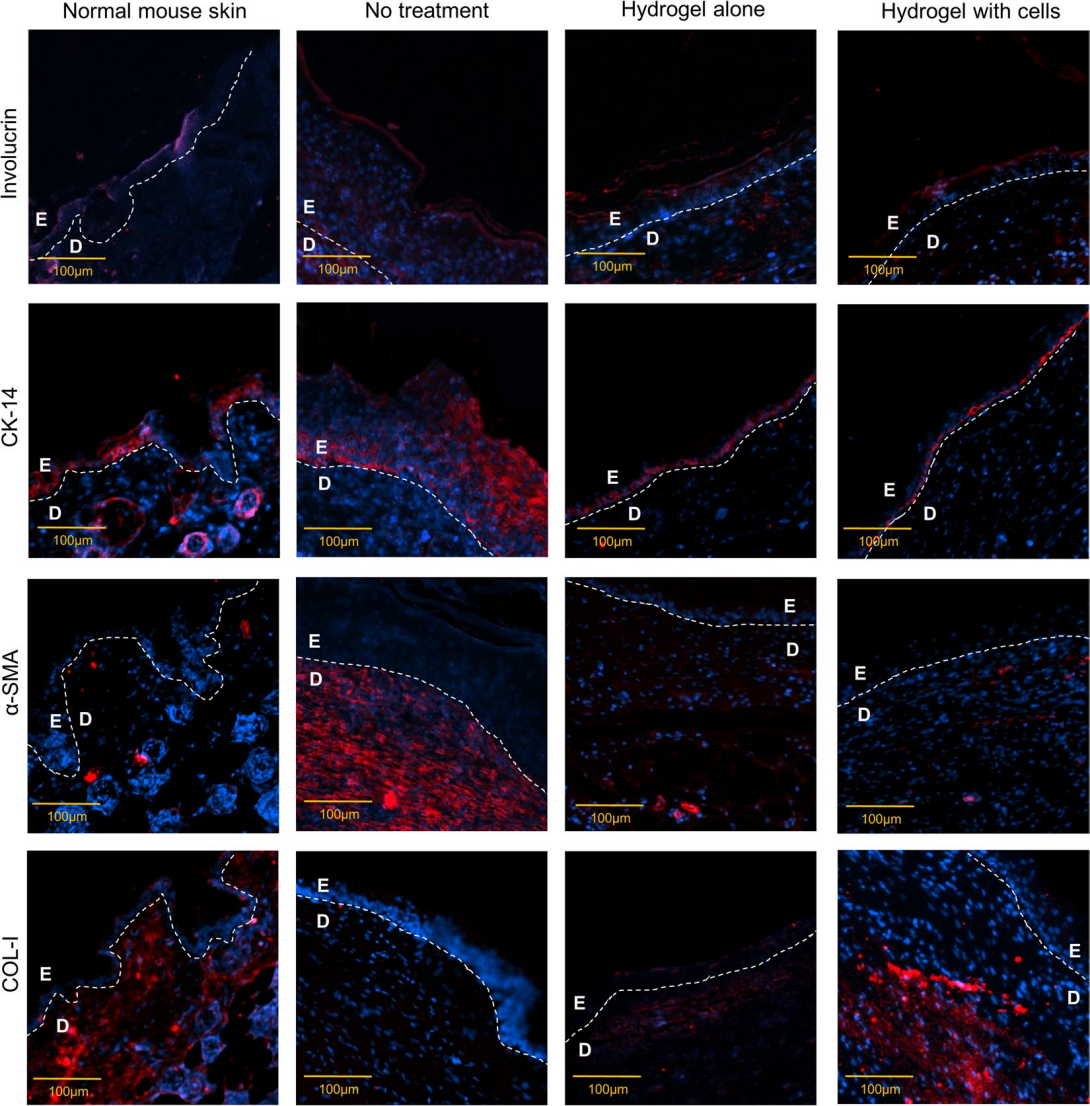
Immunohistochemical staining of involucrin, CK-14, α-SMA, and COL-I for different treatment groups under 100**×** magnification. E refers to the epidermis layer; D refers to the dermis layer. Nucleus stained blue, specific markers stained red.

At day 13, involucrin was detected at the upper layer of the epidermis, while cytokeratin 14 (CK-14) was expressed at the stratum basal layer of the epidermis across all groups analyzed, i.e., normal skin, injured skin with no treatment (NT), injured skin treated with hydrogel alone (HA), and hydrogel with cells (HC) (Fig. 6). The staining intensity of involucrin in normal mouse skin was higher than that in the other groups. High CK-14 expression was detected in the NT group, while similar expression levels were observed along the basal layer of the epidermis in the normal skin, HA and HC treatment groups.

Epidermal maturation reestablishment after tissue injury can be explored by staining for involucrin, a marker for epidermal maturation at the suprabasal layers and for CK-14, which localizes to the basal layer. In the ordinary keratinocyte maturation process, the active proliferative keratinocytes move from the stratum basal level to the top, undergoing multiple differentiation stages and end up as dead corneocytes at the stratum corneum before shedding off. The final stage of epidermal cell differentiation is the formation of cornified cell envelope to protect the corneocytes^40^. Since involucrin is a precursor protein of the cornified envelope, it is linked to the final maturation stage of the keratinocytes. A lack of involucrin expression intensity may be the result of the epidermis being still in the early maturation stage in all treated groups. CK-14 is a marker of proliferating basal keratinocytes. Higher expression of CK-14 in the NT group than in the other groups explains the presence of active keratinocytes, supporting wound healing progression.

The expression of alpha smooth muscle actin (α-SMA) in the dermis layer of the NT group was the highest, while its expression was the lowest in the normal skin. This finding is consistent with Jang *et al*.^41^ where the control group, treated with DPBS without cells, exhibited the greatest amount of α-SMA compared to fibroblasts alone, Matriderm (collagen and elastin scaffold), and Matriderm with fibroblasts treated groups. In the granulation tissue during wound healing, fibroblasts are activated to become myofibroblasts which possesses contractile ability. This contraction property is mediated by stress fibers mainly made up of α-SMA in its cytoskeleton^42^. Hence, α-SMA expression is correlated to myofibroblasts which play a major role in granulation tissue contraction. As the wound heals, myofibroblasts are then removed via apoptosis^43^. As expected, the NT groups expressed α-SMA the most as the wound healing process is much slower and still in the active proliferative phase, while α-SMA expression was the lowest in the normal skin as the wound healing process is inexistent. The number of myofibroblast in the HA and HC treatment groups may have already been reduced via apoptosis but was still relatively higher than that in the normal skin.

COL-I was highly expressed in the dermis of the normal skin since COL-I is the main structural component of the dermis^42^. Consistent with the histological findings, the wound in the NT group was probably in the proliferative phase and therefore, COL-I was weakly expressed. Indeed, during the proliferative stage, collagen type III is the major component of the granulation tissue. However, it will progressively be replaced by COL-I during the remodeling phase^29^. The expression of COL-I in the HA and HC groups matched the H & E results, suggesting that these groups are in the remodelling stage since COL-I expression is more than that in the NT group but less than that in the normal skin. Even though the IHC staining indicated that the expression of COL-I in the HA and HC treatment groups was similar, the antibody used only reacts with mouse species. Therefore, COL-I expression may be higher in the HC group as COL-I from human was not detected. This is in line with the Masson Trichrome findings where more collagen fibers were observed in the HC group compared to the HA group.

### Transmission electron microscopy (TEM)

**Figure 7:**
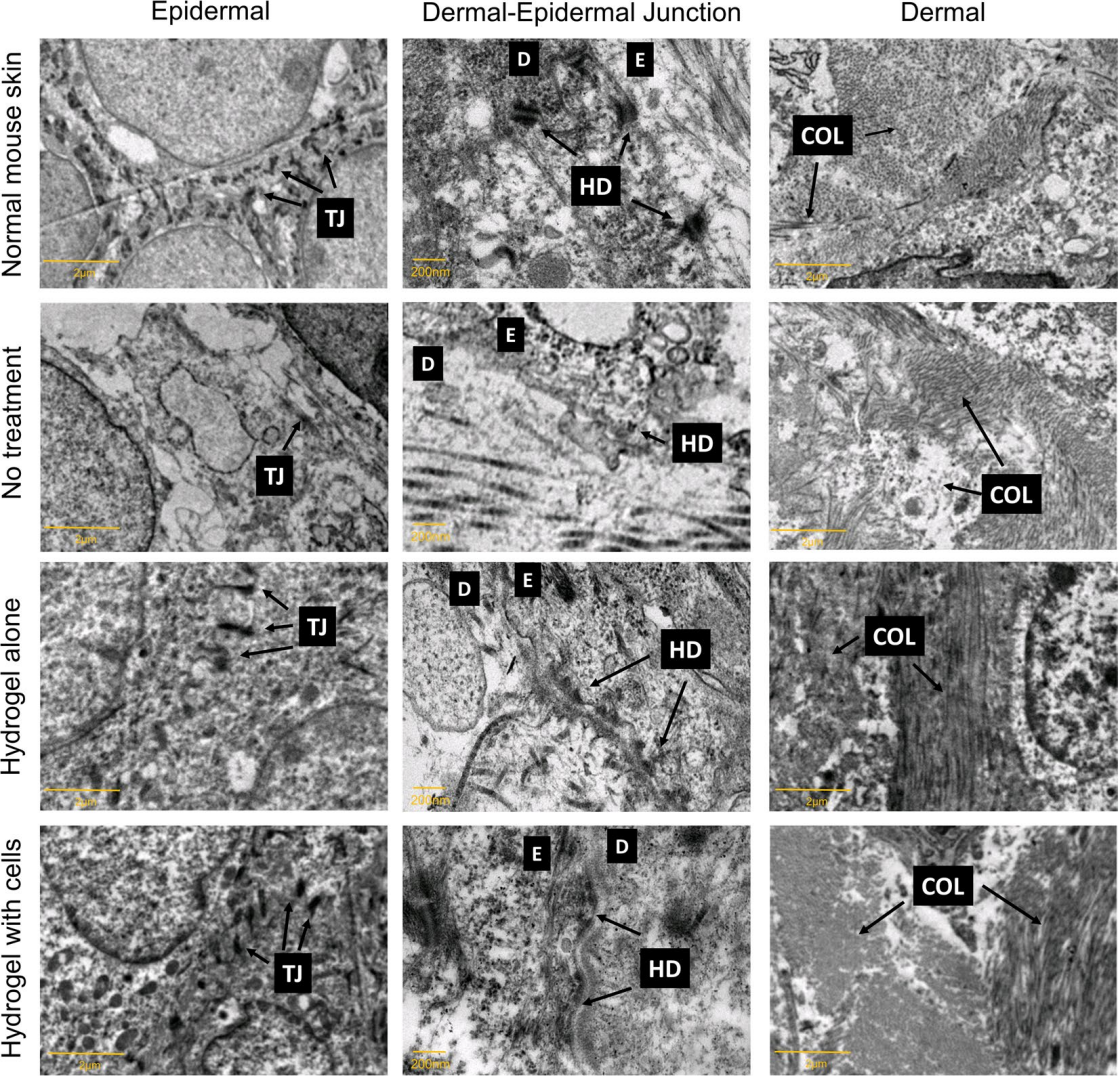
Transmission electron micrographs of the epidermal (4200**×** magnification), dermal-epidermal junction (43,000**×** magnification), and dermal structure (4200**×** magnification) for the different treatment groups. TJ refers to tight junction; HD refers to hemidesmosome; E refers to the epidermis layer; D refers to the dermis layer; COL refers to collagen fibers. Arrow for COL points to cross section and longitudinal section.

The epidermal, dermal-epidermal junction (DEJ) and dermal structure were also analyzed by using TEM. As observed in Fig. 7, for the HA and HC treatment groups, the existence of tight junctions and intermediate filaments of keratin in the epidermal layer, hemidesmosomes in the DEJ, and compact collagen fibrils in the dermal layer were almost similar to those in the normal skin. Although the NT group also showed those structures, the tight junctions in the epidermal layer were less prominent, the DEJ were not clearly defined, and the collagen fibrils were not as dense as those in the other groups, consistent with the Masson’s trichrome findings.

TEM was performed to evaluate the ultrastructure of the regenerated skin, which provides an indication of the new skin’s integrity. Integrity of the epidermis layer is evaluated by the presence of tight junctions known as desmosomes between one keratinocyte and another, while the adhesion of the epidermis to the dermis at the basal membrane is evaluated through the presence of hemidesmosomes^44^. Since the dermis layer is primarily made up of collagen, the collagen fibrils, which are present in cross section or longitudinal section, depict the integrity of the dermis^45^. The TEM results revealed that the regenerated skin’s integrity of mice in the HA and HC groups is better compared to the NT group, suggesting that the healing process is still in progress in the NT group.

In summary, all *in vivo* studies suggest that HC treatment is superior compared to NT in terms of wound healing. Even though there was no difference in wound closure rates between the NT and HA groups, histology, IHC, and TEM analysis revealed faster wound healing in the HA group. Despite the fact that most of the analysis showed similar results between the HA and HC treatment groups, the rate of wound closure was significantly higher at day 7 in the HC group. Moreover, Masson’s Trichrome staining at day 13 demonstrated the presence of more collagen fibers in the HC group, indicating a stronger dermis. Thus, these data suggest that the hydrogel which acts as a cell carrier and simultaneously as a wound dressing, is advantageous to accelerate wound healing.

Nevertheless, future work may include exploring the addition of other additives to the current BC/AA hydrogel such as dextran^46^, starch^47^, gelatin^48,49^, chitosan^50^, alginate^51^ or collagen^51^ to improve its biocompatibility and cell proliferation.

## Conclusion

In conclusion, *in vitro* studies showed that BC/AA hydrogel exhibits fast cell attachment, maintains cell viability, limits cell migration and allows cell transfer. *In vivo* studies using gross appearance, histological, IHC, and TEM analysis indicate that treatment with HA and HC accelerates wound healing compared to NT. Gross appearance and Masson’s trichrome staining demonstrated that HC treatment yielded better results than HA. Overall, these results suggest the promising application of BC/AA hydrogel as a cell carrier for the delivery of keratinocytes and fibroblasts, in addition to its function as a wound dressing, to promote full-thickness wound healing. The use of non-cultured skin cells directly, could also potentially be utilized for immediate treatment. All these factors together can dramatically shorten the time needed for treatment of large wounds.

## Materials and Methods

### Preparation of BC/AA hydrogel

BC/AA hydrogel was prepared as previously reported^15^. Briefly, the purified BC, sourced from *nata de coco*, was blended, frozen, and freeze-dried at −110 °C. It was then micronized in a variable-speed rotor mill (Pulverisette 14; Fritsch, Germany). A 1% (w/v) dispersion of BC in distilled water was mixed with AA solution (Sigma-Aldrich, Czech Republic) at 40:60 (AA:BC) ratio to produce a dispersion. The dispersion was pipette into petri dishes and exposed to 35 kGy electron beam irradiation dose at Malaysian Nuclear Agency. The hydrogel was neutralized with 1 M sodium hydroxide (NaOH) and then cut into the desired diameter with about 1 mm thickness and autoclaved (121 °C, 20 minutes).

### Cell harvest and culture of human skin

The study was approved by the Universiti Kebangsaan Malaysia Research Ethics Committee (no. FF-2015-376). Written informed consent was obtained from all subjects prior to the beginning of the study and all experiments were performed in accordance with relevant guidelines and regulations. Redundant skin tissue samples were obtained from three patients, who underwent abdominoplasty, and were processed as previously described^4^. Briefly, skin tissue samples were cleaned, minced, and digested with 0.6% collagenase type I (Worthington, USA) for four to five hours in a 37 °C incubator shaker followed by cell dissociation using 0.05% Trypsin-EDTA (Gibco, USA) for 10 minutes. Epidermal keratinocytes (EK) and dermal fibroblasts (DF) were cultured in equal amount of serum free medium Epilife (Gibco) and serum completed medium F12:DMEM (Ham’s F-12 nutrient:Dulbecco’s Modified Eagle Medium) (1:1; Gibco) supplemented with 10% fetal bovine serum (FBS) (Biowest, USA), 1% antibacterial-antimycotic (Gibco), 1% Glutamax (Gibco, USA) and 2% HEPES (Gibco) at 37 °C, 5% CO_2_. When cells reached 70–80% confluency, differential trypsinization was performed by using 0.05% Trypsin-EDTA for 3 minutes to dissociate DF from the culture plate. EK and DF were sub-cultured separately until the required number of cells was attained with medium changed every 2–3 days. Cells at passage 2 to 4 were used in all experiments.

### *In vitro* studies

#### Cell attachment

Forty thousand (4 × 10^4^) EK and DF were separately seeded on the sterilized hydrogel pre-soaked in the culture medium overnight. The cells were allowed to attach at 37 °C, 5% CO_2_. Every hour for the first six hours, the hydrogel was washed gently with Dulbecco’s Phosphate Buffered Saline (DPBS) (Sigma, USA) and the remaining cells contained in DPBS were counted by using a hemocytometer with 0.4% trypan blue solution (Sigma). The percentage of cell attachment was determined every hour for six hours and calculated as indicated below:

$${\rm{Cell}}\,{\rm{attachment}}( \% )=({\rm{Initial}}\,{\rm{cell}}\,{\rm{seeding}}-{\rm{number}}\,{\rm{of}}\,{\rm{cells}}\,{\rm{in}}\,{\rm{DPBS}})/\mathrm{Initial}\,{\rm{cell}}\,{\rm{seeding}}\times 100$$

The cells on the hydrogel were then fixed with 4% paraformaldehyde (Sigma) overnight and permeabilized with 0.5% Triton-X (Sigma) for 20 minutes followed by blocking with 10% goat serum (Invitrogen, USA) for an hour at 37 °C. Later, cells were stained with Phalloidin CF 488 A conjugate (Biotium, USA) diluted 1:80 in DPBS and DAPI (Invitrogen) diluted 1:15,000 in DPBS. Fluorescence imaging was performed by using a Nikon A1R confocal microscope (Nikon, Japan).

#### Cell viability

Forty thousand (4 × 10^4^) EK and DF were separately seeded on the hydrogel and on polystyrene cell culture plates. At day 1 and 3, the cells were stained with LIVE/DEAD® Viability/Cytotoxicity Kit for mammalian cells (Invitrogen) according to the manufacturer’s protocol. Briefly, a working solution containing 2 µM calcein AM and 4 µM ethidium homodimer 1-red (EthD-1) in DPBS was prepared. Cells were washed with DPBS and then incubated with the working solution. After 30 minutes, cells were observed by using a Nikon A1R confocal microscope (Nikon).

#### Cell proliferation

Ten thousand (1 × 10^4^) EK and DF were separately seeded on the hydrogel in a 48 well plate. Vybrant® MTT Cell Proliferation Assay Kit (Invitrogen) was conducted according to the manufacturer’s recommendation. At day 1 and 7, the hydrogel was transferred to a new culture well to eliminate the possibility of false positive by cells that may have attached on the well. Spent medium was replaced with 100 µL fresh medium of Epilife or F12:DMEM. Ten microliters (10 µL) of MTT were added into every well and cells were incubated at 37 °C for 4 hours. Hundred microliters (100 µL) of SDS-HCl solution were then added and after another 4 hours of incubation, the solution was transferred to a 96 well plate and absorbance was read at 570 nm using a spectrophotometer reader (Bio-Tek, Power Wave XS, USA).

#### Cell morphology

Forty thousand (4 × 10^4^) EK and DF were seeded separately on the hydrogel. At Day 1 and 7, the construct was fixed with 4% glutaraldehyde (Sigma) and then dehydrated through serial dilutions of ethanol and dried in a critical point dryer (Leica EM CPD300, Germany). The samples were sputter-coated with gold and observed by scanning electron microscope (SEM) (LEO 1450 VPSEM, Zeiss, Germany).

#### Cell migration

To distinguish EK and DF, the cytoplasm of EK was stained with green cell tracker CMFDA (Invitrogen), while the nucleus of DF was stained with Hoechst Blue Dye (Invitrogen) according to the manufacturer’s instructions at a final concentration of 5 µM. Forty thousand (4 × 10^4^) EK and an equal amount of DF were seeded on the hydrogel followed by 3D confocal imaging on day  0, 3 and 7 using a Nikon A1R confocal microscope (Nikon).

#### Cell transfer

Ovine collagen hydrogel (OCH; Tissue Engineering Centre, Universiti Kebangsaan Malaysia) was used to represent the native skin. The ovine collagen solution was prepared as described by Fauzi *et al*.^17^. The ovine collagen solution was neutralized with 1 M NaOH and allowed to polymerize at 37 °C to form the hydrogel.

Eighty thousand (8 × 10^4^) EK and an equal amount of DF were stained as mentioned in the section above, followed by seeding on the hydrogel. After 4 hours, the hydrogel surface which contained the skin cells, was flipped onto the OCH. At day 1 and 3, the BC/AA hydrogel was peeled off from the OCH and the cells on each of the individual hydrogel were captured. The total number of cells at three different locations on the BC/AA hydrogel and OCH were determined by using Image-J software (version 1.50i). The percentage of cells observed by using a Nikon A1R confocal microscope (Nikon) was calculated as indicated below:

$${\rm{Percentage}}\,{\rm{of}}\,{\rm{cells}}=X/Y\times 100$$

where X = Total number of cells at 3 locations on BC/AA hydrogel or OCH; Y = Total number of cells at 3 locations on BC/AA hydrogel and OCH

### *In vivo* studies

#### Establishment and treatment of full-thickness wound

Animal studies were approved by Universiti Kebangsaan Malaysia Animal Ethics Committee (no. FF/2015/CAIRUL/29-SEPT./703-OCT.-2015-JAN.-2016) and all experiments were performed in accordance with relevant guidelines and regulations. Nine male athymic mice, 8 weeks old, weighing between 20–25 g were anesthetized intramuscularly by a mixture of xylazine (25 mg/mL), ketamine (25 mg/mL) and zoletil (12.5 mg/mL) at a 1:1:1 volume ratio with 0.1 mL volume per 25 g body weight. After the area was cleaned and disinfected using 70% ethanol, a 0.79 cm^2^ full-thickness skin wound was created at both sides of the hind legs.

The mice were separated into three different groups (N = 6/group). Group 1 received no treatment, group 2 and 3 were treated with hydrogel alone without human skin cells and hydrogel with human skin cells, respectively. For group 3, mixed skin cells containing forty thousand (4 × 10^4^) EK and sixty thousand (6 × 10^4^) DF were seeded on the hydrogel for four hours before flipping the hydrogel onto the wound site. Both non-seeded and seeded hydrogel were placed inside the wound. All wounds were covered with OtoSilk sutured to the mice skin using degradable suture followed by wound dressing. All mice were housed in an individual ventilated cage system (Biobubble, USA). On day 13, the tissue at the wound area was harvested for histopathological, immunohistochemical (IHC) and transmission electron microscopy (TEM) evaluation.

#### Visual observation of wound closure

The wound area was grossly observed at day 0, 7, 9, 11, and 13 with a measurement scale by using a digital camera. The area of the wound was measured by utilizing Image-J software and wound closure at each time point was calculated as follow:

$$ \% \,{\rm{wound}}\,{\rm{closure}}=({\rm{Initial}}\,{\rm{wound}}\,{\rm{area}}-{\rm{Wound}}\,{\rm{area}}\,{\rm{at}}\,{\rm{the}}\,{\rm{time}}\,{\rm{of}}\,{\rm{observation}})/\mathrm{Initial}\,{\rm{wound}}\,{\rm{area}}\times 100$$

#### Histological analysis

The harvested wound tissues were fixed with 10% buffered formalin and embedded in paraffin blocks. The samples were cut into 5 µm sections with a microtome (Leica, Germany). After dewaxing with a series of xylene and alcohol, sections were stained with H&E and Masson’s trichrome stain followed by observation under a light microscope. The thickness of the epidermis and dermis layer of the regenerated wound was measured with the assistance of Image-J software at 10 different random locations for each micrograph.

#### Immunohistochemistry (IHC) staining

For IHC, tissue sections on the slides were dewaxed with a series of xylene and alcohol and treated with antigen retrieval solution, i.e., citrate buffer pH 6 (Sigma). Tissue sections were incubated with 10% goat serum for 1 hour at 37 °C, followed by incubation with primary antibodies i.e., involucrin, CK-14, α-SMA, and COL-I (Abcam, UK) overnight at 4 °C. The next day, tissue sections were incubated with secondary antibodies (Invitrogen) for 2 hours at 37 °C and counterstained with DAPI for 20 minutes at room temperature. The details of the primary and secondary antibodies used are provided in Table 1. Observation was performed under Nikon A1R confocal microscope (Nikon).

**Table 1:** List of primary and secondary antibodies used.

Primary antibody	Dilution	Target species	Secondary antibody	Dilution
Rabbit polyclonal to involucrin (ab53112)	1:50	Mouse, human	Alexa Fluor^®^ 594 goat anti-rabbit IgG	1:250
Rabbit monoclonal to CK-14 (ab181595)	1:1000	Mouse, human	Alexa Fluor^®^ 594 goat anti-rabbit IgG	1:250
Mouse monoclonal to α-SMA (ab7817)	3:50	Mouse, human	Alexa Fluor^®^ 594 goat anti-mouse IgG	1:250
Rabbit polyclonal to COL-I (ab21286)	1:250	Mouse	Alexa Fluor^®^ 594 goat anti-rabbit IgG	1:250

#### Transmission electron microscopy (TEM)

Tissue blocks of 1 mm were fixed in 4% glutaraldehyde in 0.1 M phosphate buffer and post-fixed with 1% osmium tetroxide for 2 to 4 hours. *En bloc* staining was performed with aqueous uranyl acetate for 1 hour, followed by dehydration and embedding with 100% resin in beam capsule. The tissue blocks were cut into 0.5–1.0 µm thick sections and stained with toluidine blue before proceeding to ultrathin sectioning (70 nm). The sections on the grid were impregnated with uranyl acetate and lead citrate and viewed under a transmission electron microscope (FEI Tecnai G2 Spirit Biotwin, USA).

### Statistical analysis

For multiple group comparison, statistical analysis was performed by using one-way ANOVA followed by post-hoc Tukey, while, for comparison between two groups, Student t-test was carried out by using Graph Pad Prism version 6.0. P values < 0.05 were considered statistically significant. All quantitative data values for *in vitro* studies were obtained from triplicates (N = 3) while six replicates (N = 6) were used for *in vivo* studies. All values are expressed as the means ± standard deviations (SD).

### Data availability

All data generated or analyzed during this study are included in this published article.
